# Swimming performance of a pelagic species in the Yangtze River under different exposure modes of the total dissolved gas supersaturation

**DOI:** 10.1093/conphys/coac047

**Published:** 2022-07-21

**Authors:** Qianfeng Ji, Kefeng Li, Yuanming Wang, Ruifeng Liang, Jingjie Feng, Ran Li, David Z Zhu

**Affiliations:** State Key Laboratory of Hydraulics and Mountain River Engineering, Sichuan University, Yihuan Road, Chengdu 610065, China; Department of Civil and Environmental Engineering, University of Alberta, Edmonton, Alberta, T6G 1H9, Canada; State Key Laboratory of Hydraulics and Mountain River Engineering, Sichuan University, Yihuan Road, Chengdu 610065, China; State Key Laboratory of Hydraulics and Mountain River Engineering, Sichuan University, Yihuan Road, Chengdu 610065, China; State Key Laboratory of Hydraulics and Mountain River Engineering, Sichuan University, Yihuan Road, Chengdu 610065, China; State Key Laboratory of Hydraulics and Mountain River Engineering, Sichuan University, Yihuan Road, Chengdu 610065, China; Department of Civil and Environmental Engineering, University of Alberta, Edmonton, Alberta, T6G 1H9, Canada

**Keywords:** water temperature, total dissolved gas supersaturation, swimming performance, pelagic fish, Exposure time

## Abstract

During flood discharges of upstream dams in the Yangtze River, the pelagic fish have a stress risk from total dissolved gas (TDG) supersaturation in the river water. This study took the silver carp as the object and systematically evaluated the effects of TDG supersaturation levels and exposure time on their critical swimming speed (Ucrit) at different temperatures. The external symptoms of gas bubble disease were found when TDG levels exceeded 130%. Both exposure time and TDG level did not significantly impact the Ucrit of fish under 6 days of non-lethal exposure (110%, 120%, 130% TDG) with lower or higher water temperature. Significant differences in Ucrit were found among different exposure times at 11.0 ± 1.0°C under 10 hours of lethal exposure (135%, 140%, 150% TDG) and the Ucrit reduced by 59.88%, 83.32%, and 92.40%, respectively. TDG level had a significant impact on the Ucrit at 21.0 ± 1.0°C when exposure time exceeded 8 hours. Ucrit at 21.0 ± 1.0°C water were significantly greater than those at 11.0 ± 1.0°C water where conditions had the same TDG supersaturation and exposure time. Differences in Ucrit between temperatures ranged from 3.24 to 6.12 BL/s under non-lethal exposure and from 6.38 to 13.88 BL/s under lethal exposure. The results of this study can provide a reference for fish conservation during flood discharge.

## Teaser

This study systematically evaluated the swimming performance of a pelagic fish species in the Yangtze River under different exposure modes of total dissolved gas (TDG) supersaturation. The lethal exposure significantly deteriorated the Ucrit of silver carps while the non-lethal exposure did not. Water temperature also impacted fish’s swimming ability in TDG supersaturated water. The results can provide a reference for fish conservation during flood discharge.

## Introduction

Dams are constructed for multiple purposes including flood control, power generation, water supply and navigation (Jumani *et al.*, 2020). A total of 43 400 dams had been built in the Yangtze River basin in China by 2019, causing 333 rivers cutoff with a total length of 1017 km (National Audit Office of the People’s Republic of China, 2018). These dams transformed rivers from a natural ecological corridor mode into a fragmented reservoir-dam-reservoir mode, severely disrupting the continuity of aquatic habitats (Hoeinghaus, 2018; Yang *et al.*, 2019). A series of ecological and environmental problems have been caused by the construction and operation of dams (Grill *et al.*, 2019; Zhang *et al.*, 2020). During the dam discharge period, a large amount of water flow is spilled into the stilling basin and can result in the significance of air entrainment and its dissolution, which leads to the supersaturated total dissolved gases (TDGs) in the downstream river (Lu *et al.*, 2019). Gas bubbles released from the supersaturated TDG water are always attached to the body surface of fish, which affects their swimming performance (Gunnarsli *et al.*, 2009; Yuan *et al.*, 2021). At the same time, the gases enter the fish’s bodies when they breathe, participate in the blood circulation and, finally, cause embolism in fish’s organs. It is widely known as gas bubble disease (GBD) (Skov *et al.*, 2013; Pleizier *et al.*, 2020a; Ji *et al.*, 2021). The mortalities of fish will occur when GBD develops to a certain extent (Weitkamp and Katz, 1980; Weitkamp, 2008).

Swimming is one of the basic abilities of fish to survive, which is used for migration, predation and avoidance of natural enemies (Yuan *et al.*, 2021). The critical swimming speed (Ucrit) is a standard measurement to assess the swimming ability of fish and reflects the prolonged and aerobic swimming performance (Plaut, 2001). Fish usually use the Ucrit for their routine activities such as foraging and holding (Katopodis and Gervais, 2012; Cano-Barbacil *et al.*, 2020). A few studies showed that TDG supersaturation can affect the swimming performance of fish (Wang *et al.*, 2017 and 2018). Once fish suffer from GBD, bubbles accumulating in the fins affect their balance and movement ability and bubbles released from the gill filament block blood vessels and weaken respiration and oxygen delivery. Gas embolism and congestion in the fish’s muscles also reduce their athletic ability (Weitkamp and Katz, 1980; Weitkamp, 2008). During the flood season, the frequent water discharge from dams can cause TDG supersaturation in the downstream river (Ma *et al.*, 2018). Fish in dam-regulated rivers may encounter water with supersaturated TDG, which can reduce the swimming ability of these fish and finally affect their routine activities (Wang *et al.*, 2018; Pleizier *et al.*, 2020a).

TDG is not always supersaturated in the vertical direction as the water depth increases because hydrostatic pressure could limit the formation of gas bubbles and the compensation rate is about 10% per meter depth (Pleizier *et al.*, 2020b; Wang *et al.*, 2020). It means that pelagic fish face a greater risk of supersaturated TDG stress than benthic species when water is discharged from dams. Previous studies mainly focused on the swimming performance of salmon in North America or the benthic species in the Yangtze River, while only limited work has been conducted to explore the effects of TDG on the swimming performance of pelagic species (Schiewe, 1974; Dawley and Ebel, 1975; Wang *et al.*, 2017 and 2018).

Many dams have been built in the upper and middle reaches of the Yangtze River. The water temperature is usually 10–15°C in the summer in the upper Yangtze River where the altitude is above 2000 m (He *et al.*, 2016), while it is 20–25°C in the middle Yangtze River (Tao *et al.*, 2019). The levels of TDG supersaturation downstream of dams in the Yangtze River basin usually range from 100% to 150% during flood discharge (Feng *et al.*, 2010). As one of the four major Chinese carp species, the silver carp *(Hypophthalmichthys molitrix)* is a pelagic fish that is widely distributed in the Yangtze River basin (Duan *et al.*, 2009; Shen and Gao, 2012). The swimming performance of silver carps in flood season may be susceptible to supersaturated TDG. Experiments on the swimming performance of silver carps were carried out in this study to explore the influence of supersaturated TDG on the swimming performance of silver carps. The results of this study can help to provide a reference for the protection of pelagic fish inhabited in the Yangtze River basin.

## Materials and methods

### Ethical statement

The animal study proposal was approved by the Ethics Committee for Animal Experiments of Sichuan University (No. 2019062101). All experimental procedures were performed in accordance with the Regulations for the Administration of Affairs Concerning Experimental Animals approved by the State Council of the People’s Republic of China.

### Experiment fish

Half-year-old silver carps provided by the Fisheries Institute of the Sichuan Academy of Agricultural Sciences were used in this study. The experiment of the lower-temperature group was carried out between 4 and 31 December 2019, while the experiment of the higher-temperature group was carried out between 10 and 25 July 2020. Water temperature during these two periods only fluctuated within ±1.0°C. The silver carps were immediately acclimated in four tanks (length × width × depth: 1.2 × 1 × 1.2 m) with freshwater for 48 hours after being transported to the laboratory. Freshwater in tanks came from aerated tap water and replaced half once a day. The dissolved oxygen (DO) concentration, TDG level and temperature of holding water were 9.6–10.2 mg/l, 100–101% and 11.0 ± 1.0°C, respectively, during the lower-temperature experiment and were 7.7–8.4 mg/l, 91–93% and 21.0 ± 1.0°C, respectively, during the higher-temperature experiment. Other water quality parameters met the national standard for tap water quality of the People’s Republic of China. Fish were fasted during acclimation and experiment.

### Experimental design

A total of 940 silver carps were used to explore their swimming performance under conditions of two water temperatures with six TDG supersaturation levels. Two water temperature scenarios were selected in this study, 11.0 ± 1.0°C and 21.0 ± 1.0°C, which are based on the water temperature in the Yangtze River during flood season.

**Figure 1:**
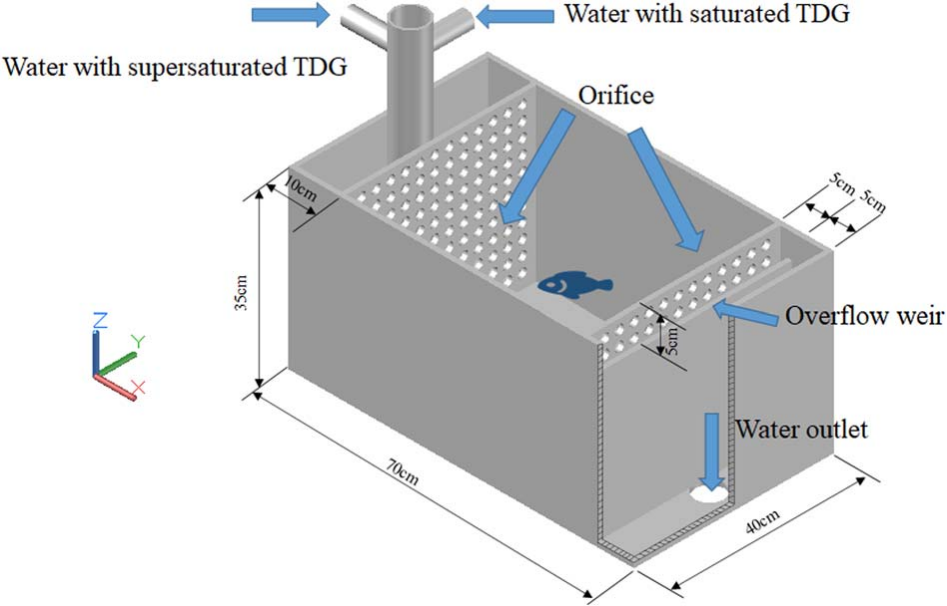
The flow-through tank used for fish exposure. The expected TDG level can be achieved by adjusting the mixing rate of aerated tap water and TDG supersaturated water. The TDG supersaturated water can smoothly flow in and out of the tank through the orifice plate.

The water with supersaturated TDG was generated by a TDG supersaturation system, which was described in detail by Wang *et al.* (2015). Briefly, tap water and air were introduced into a pressure vessel through a pump and an air compressor, respectively. Excess air was dissolved under high pressure, which resulted in TDG supersaturation. There were six TDG supersaturation levels selected in this study. According to previous research, mortality rarely occurred when juvenile silver carps were exposed to the water where TDG supersaturation was no more than 130% (Ji *et al.*, 2021). Thus, three TDG levels of 110%, 120% and 130% were designated as non-lethal exposure groups to study the swimming ability of fish under non-lethal exposure. And three TDG levels of 135%, 140% and 150% were designated as lethal exposure groups to study the swimming ability of fish under lethal exposure. As shown in Fig. 1, TDG supersaturated water was introduced to flow-through tanks for fish exposure. The TDG level was kept by adjusting the open degree of valves for aerated tap water (100% TDG) and TDG supersaturated water. TDG supersaturation levels were measured by a TDG pressure meter (PT4 TRACKER, Point Four Systems Inc., Canada) calibrated before the experiment.

**Figure 2:**
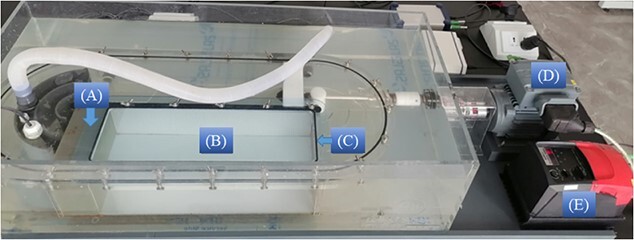
Picture of the swimming tunnel respirometer used in this study: (**A**) the honeycomb screen, (**B**) the swimming chamber, (**C**) the wire mesh, (**D**) the electric motor and (**E**) the frequency converter.

The duration of non-lethal exposure was 6 days because the flood discharge duration of cascade reservoirs in the upper Yangtze River usually does not exceed a week (Ma *et al.*, 2018), and 50 juvenile silver carps were used for each non-lethal exposure group. The swimming ability of juvenile silver carps were measured every 24 hours, and 7 fish were measured each time to get their average swimming speed. The duration of lethal exposure was set as 10 hours because of the rapid fish mortality under lethal exposure (Ji *et al.*, 2021). One hundred juvenile silver carps were used for each lethal exposure group. The swimming speed of juvenile silver carps was measured every 2 hours, and seven fish were measured each time to get their average swimming speed. As a comparison, swimming speed at 100% saturation (without TDG supersaturation exposure) was measured with seven juvenile silver carps at the beginning of TDG exposure for each mode. The information on fish in each group is shown in Table 1, and the sizes of fish used at different TDG levels were similar for the same exposure mode except for the non-lethal exposure group with the temperature of 11.0 ± 1.0°C (least significant difference for weight: *df* = 3, *F* = 11.337, *P* = 0.000; for length: *df* = 3, *F* = 12.371, *P* = 0.000). Each fish was used only once for Ucrit test.

**Table 1:** Detailed information of silver carps used in the swimming ability test group

Temperature (°C)	Exposure mode	TDG supersaturation	Number of fish	Weight (g)	Length (cm)
		100%	10	4.95 ± 0.37^bc^	7.71 ± 0.19^bc^
	Non-lethal exposure	110%	50	5.84 ± 0.27^ab^	8.12 ± 0.11^ab^
		120%	50	4.45 ± 0.23^c^	7.48 ± 0.12^c^
11.0 ± 1.0		130%	50	6.33 ± 0.22^a^	8.38 ± 0.10^a^
		100%	10	1.55 ± 0.13	5.37 ± 0.13
	Lethal exposure	135%	100	1.56 ± 0.07	5.17 ± 0.08
		140%	100	1.51 ± 0.06	5.05 ± 0.07
		150%	100	1.54 ± 0.07	5.20 ± 0.07
		100%	10	3.32 ± 0.17	6.86 ± 0.18
	Non-lethal exposure	110%	50	3.41 ± 0.10	6.96 ± 0.06
		120%	50	3.31 ± 0.11	6.87 ± 0.08
21.0 ± 1.0		130%	50	3.49 ± 0.15	6.89 ± 0.13
		100%	10	1.47 ± 0.10	5.12 ± 0.11
	Lethal exposure	135%	100	1.59 ± 0.07	5.10 ± 0.06
		140%	100	1.42 ± 0.06	5.10 ± 0.06
		150%	100	1.54 ± 0.05	5.15 ± 0.05

Data on temperature, body length and weight are expressed as Mean ± SE. The superscript letters after the values represent results from a post hoc multiple comparison test, which means not sharing a common lowercase letter are significantly different (*P* < 0.05).

### Measurement of swimming ability

A swimming tunnel respirometer (Loligo Systems SW10150, Denmark) was used to measure the Ucrit. It had a total water volume of 30 l and the swimming chamber was 9 l (length × width × height: 46 × 14 × 14 cm) (Fig. 2). A honeycomb screen was fixed upstream of the swimming chamber to reduce the turbulence and ensure a uniform flow velocity in the swimming chamber. A wire mesh was fixed downstream of the swimming chamber to prevent fish from escaping. Water flow was driven by a propeller powered by an electric motor and the flow velocity was controlled by adjusting the motor frequency. The water used for the swimming speed test was from aerated tap water with 100% TDG. At each time point, seven fish were simultaneously introduced to the swimming chamber to test their Ucrit. The cross-sectional area of seven fish was < 10% of the swimming chamber so the mutual influence of fish and the fish obstruction of flow could be negligible (Pang *et al.*, 2013; Cai *et al.*, 2014).

Ucrit was determined using the increasing velocity method (Wang *et al.*, 2017). Fish were put in the swimming chamber under a 5-cm/s flow velocity for 20 minutes to eliminate the effect of the transfer process. In the swimming chamber, flow velocity was then steadily increased at an increment of *Δv* = 10 cm/s every 20 minutes from 5 cm/s until the fish was exhausted and leaned against the downstream wire mesh for more than 2 minutes. The Ucrit was calculated as follows:

$$ Ucrit=v+\left(T/\varDelta T\right)\times \varDelta v, $$

where *v* is the secondary maximum velocity when the fish was exhausted, *Δv* is the flow velocity increment (10 cm/s in the present study), *ΔT* is the duration of each flow velocity (20 min in the present study) and *T* is the length of time that fish swam at the maximum flow velocity before they were exhausted.

### Statistical analysis

All the swimming speeds were presented as relative values (BL/s) calculated by dividing the absolute value by the body length. The effect of TDG level, exposure time and their interaction on swimming speeds of fish was determined by using two-way ANOVA analysis, which was followed by a post hoc multiple comparison test (least significant difference) to determine the difference between the values at different treatment group. The independent samples t-test method was used to compare the differences between two temperatures. Ucrit and body size data fulfilled the homogeneity of variances (tested with Levene’s test). The significant difference level was set at *P* < 0.05.

## Results

### Abnormal behavior and GBD of fish during exposure

**Figure 3:**
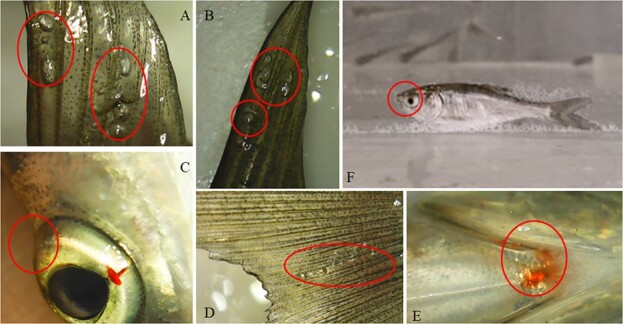
The GBD of silver carps under lethal exposure. (**A**) bubbles in the pectoral fin; (**B**) bubbles in the dorsal fin; (**C**) exophthalmia in the eye; (**D**) bubbles in the caudal fin; (**E**) congestion near the jaw; and (F) bubble attachment on the body surface.

In the non-lethal exposure groups, no fish died of supersaturated TDG and they swam randomly. Gas bubbles from the water adhered to the surface of fish, but they did not induce abnormal behavior of fish. However, fish mortality occurred during lethal exposure, and the dead fish showed obvious bubble disease (Fig. 3A–E). Before death, fish firstly appeared sprint swimming, then lost balance and floated sideways on the water (Fig. 3F).

### Swimming performance of fish under non-lethal exposure

**Figure 4:**
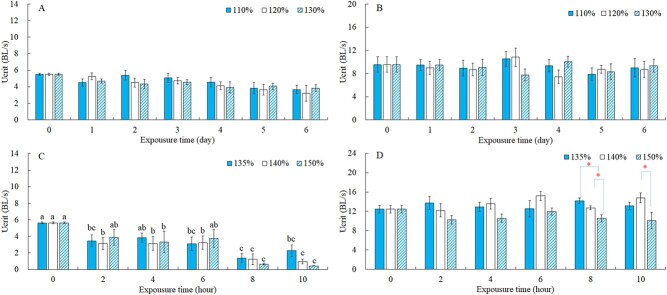
The Ucrit of fish under different experimental conditions. The values of Ucrit are shown as Mean ± SE. (**A**) and (**B**) are the results of non-lethal exposure at 11 ± 1.0°C and 21 ± 1.0°C, respectively, while (**C**) and (**D**) are the results of lethal exposure at 11 ± 1.0°C and 21 ± 1.0°C, respectively. Letters or asterisks above the bars indicate the results of a post hoc multiple comparison test (least significant difference test). For the same TDG supersaturation exposure, Ucrit at different exposure times that do not share a common letter are significantly different (*P* < 0.05). For the same exposure time, an asterisk represents a significant difference between different TDG levels (*P* < 0.05).

As shown in Fig. 4A, the Ucrit changed slightly over time under the non-lethal exposure of supersaturation TDG at 11.0 ± 1.0°C. The Ucrit of silver carps without exposure (100% TDG) was 5.51 BL/s at 11.0 ± 1.0°C, and decreased to 3.67 BL/s, 3.2 BL/s, 3.83 BL/s after exposure of 110%, 120% and 130%, respectively, for 6 days. Although the result of two-way ANOVA showed that differences in Ucrit among exposure times were significant (Table 2; *P* < 0.001), the post hoc multiple comparison test did not find any significant differences at different exposure times for each TDG level. Effects of TDG levels and the interaction between TDG level and exposure time on Ucrit were not significant (*P* > 0.05).

**Table 2:** The result of two-way ANOVA analysis for each exposure condition

Exposure mode	Temperature	Factors	*df*	*F*	*P*
		Exposure time × TDG	12	0.449	0.940
	11.0 ± 1.0	Exposure time	6	5.208	0.000
Non-lethal exposure		TDG	2	0.488	0.615
		Exposure time × TDG	12	0.572	0.861
	21.0 ± 1.0	Exposure time	6	0.462	0.835
		TDG	2	0.075	0.928
		Exposure time × TDG	10	0.486	0.896
	11.0 ± 1.0	Exposure time	5	18.060	0.000
Lethal exposure		TDG	2	0.552	0.578
		Exposure time × TDG	10	1.143	0.338
	21.0 ± 1.0	Exposure time	5	0.433	0.824
		TDG	2	10.232	0.000

The significant difference level was set at *P* < 0.05.

**Figure 5:**
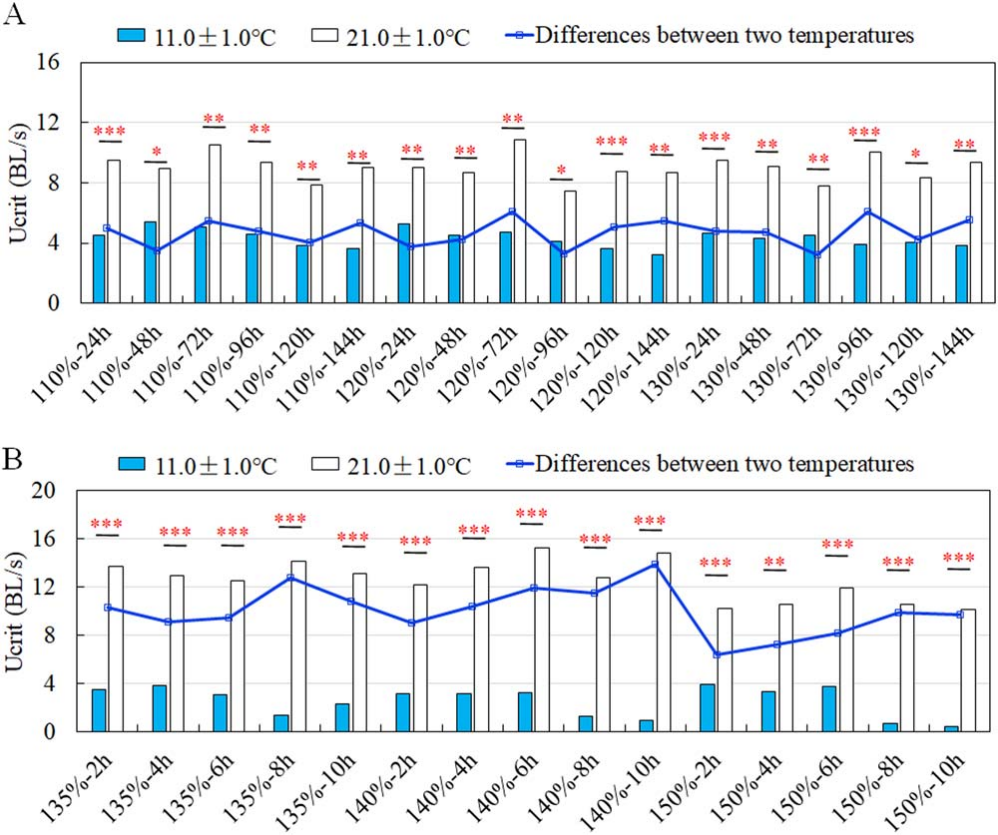
The comparisons in Ucrit between 11 ± 1.0°C and 21.0 ± 1.0°C under non-lethal (**A**) and lethal (**B**) TDG supersaturation exposure. Each condition is indicated as TDG – exposure time. The black broken line represents the difference in Ucrit between two temperatures under different exposure conditions. The significant difference between the two groups was indicated by asterisks. (^*^P < 0.05; ^**^P < 0.01; ^***^P < 0.001).

The Ucrit ranged from 7.90 to 10.54 BL/s, 7.45 to 10.85 BL/s and 7.78 to 10.05 BL/s respectively under non-lethal exposure of 110%, 120% and 130% TDG supersaturation at 21.0 ± 1.0°C (Fig. 4B). As shown in Table 2, the TDG level, exposure time and their interaction did not significantly affect the Ucrit (*P* > 0.05). The Ucrit at 21.0 ± 1.0 C was 3.24–6.12 BL/s greater than that at 11.0 ± 1.0 C under non-lethal exposure. The differences in Ucrit were significant between two temperatures under the same non-lethal exposure conditions (Fig. 5A; *P* < 0.05).

### Swimming performance of fish under lethal exposure

The Ucrit under the lethal exposure of supersaturation TDG at 11.0 ± 1.0°C showed a downward trend over time (Fig. 4C). Significant differences in Ucrit were only found among exposure times (Table 2). The results of post hoc multiple comparison test showed significant decreases in Ucrit occurred at the 2nd hour of exposure for 135% and 140% and 4th hour of exposure for 150% TDG (least significant difference for 135%: *df* = 5, *F* = 5.388, *P* = 0.001; for 140%: *df* = 5, *F* = 7.201, *P* = 0.000; for 150%: *df* = 5, *F* = 6.398, *P* = 0.000). Compared with the control group, the Ucrit reduced by 59.88%, 83.32% and 92.40% respectively after 10 hours of exposure under 135%, 140% and 150% TDG supersaturation.

The Ucrit ranged from 12.56 to 14.19 BL/s, 12.20 to 15.22 BL/s and 10.12 to 11.96 BL/s respectively under 135%, 140% and 150% TDG supersaturation at 21 ± 1.0°C (Fig. 4D). The differences in Ucrit were only significant among TDG levels (Tab. 2) and significant differences in Ucrit among TDG levels occurred at the 8th hour and 10th hour of exposure (least significant difference for 8th hour: *df* = 2, *F* = 9.724, *P* = 0.001; for 10th hour: *df* = 2, *F* = 3.656, *P* = 0.046). There existed 6.38–13.88 BL/s differences between two temperature treatments under lethal exposure, and the Ucrit at 21.0 ± 1.0°C were significantly greater than those at 11.0 ± 1.0°C under all lethal exposure conditions (Fig. 5B; *P* < 0.01).

## Discussion

The present study investigated the effect of supersaturated TDG on the Ucrit of silver carps under different exposure modes. The non-lethal exposure of TDG supersaturation did not significantly impact the Ucrit at both lower and higher temperatures. Under the lethal exposure mode, the exposure time had a marked effect on Ucrit at the lower water temperature while the TDG level played a dominant role on Ucrit at the higher water temperature. The interaction of TDG and exposure time on swimming speeds was not significant for each exposure mode. Increasing water temperature could significantly improve the Ucrit of fish in TDG supersaturated water.

## GBD of fish exposed to supersaturated TDG

Supersaturated TDG induced by dam discharge usually causes GBD of fish, which has been reported in many rivers (Ji *et al.*, 2019; Agostinho *et al.*, 2021). The TDG supersaturation in the downstream of high dams usually ranges from 100% to 150% during flood discharge (Weitkamp and Katz, 1980; Weitkamp, 2008; Qu *et al.*, 2011). The TDG supersaturation releases much slowly and continuously stresses fish in a large space for a long time (Feng *et al.*, 2010). Once the TDG level reaches a certain threshold, the fish die quickly in a short time accompanied by the symptoms of acute bubble disease (Ji *et al.*, 2021). In the present study, when the TDG level was >130%, fish mortality occurred. Under lethal exposure, a large number of bubbles attached to the surface of the fish and the stressed fish eventually floated on the surface until death. Fish suffering from GBD usually show bubble accumulation and congestion in fins and gills, bulging and bleeding eyes (Fig. 2). GBD made fish lose balance and induced abnormal behavior under lethal exposure (Espmark *et al.*, 2010; Ji *et al.*, 2021). Fish were found sprinting occasionally from a clam condition to get rid of bubbles in their bodies (Oertel, 2010).

## Effect of water temperature on Ucrit

Water temperature is one of the determinative factors affecting the physiological function and behavioral activities of aquatic organisms (Temple and Johnston, 1997; Lee *et al.*, 2003). Water temperature varies in different seasons and river reaches. Changes in water temperature have a significant influence on the swimming performance of fish—subsequently further affecting fish basic abilities such as predation, avoidance, and migration (Claireaux *et al.*, 2006). With the increase of water temperature, the mitochondrial function of fish muscle cells is enhanced and the output power of muscle tissue is increased (Guderley, 2004). Besides, the viscosity of water would decrease with the increase of water temperature, which significantly decreases the swimming resistance of fish (Bolton and Havenhand, 2005). The variation of the Ucrit usually shows a bell-shaped curve or linear increasing trend with water temperature in a certain range, which means the Ucrit of fish exhibit an increasing trend with water temperature below the optimum range (Zeng *et al.*, 2009; Pang *et al.*, 2013; Cai *et al.*, 2014). Opuszyňski *et al.* (1989) found the thermal optimum for silver carps is between 25°C and 30°C. In the present study, the water temperature did not reach the optimum range of silver carps and the Ucrit at the lower water temperature was found significantly smaller than that at the higher water temperature, which agreed with the previous studies.

## Effect of supersaturated TDG on Ucrit

The differences in Ucrit among TDG levels or exposure times were not significant under non-lethal exposure in this study (Fig. 4A and B). A similar result was also found in steelhead trout (*Steelhead, Salmon gairdneri*) whose swimming performance under the stress of 105–125% TDG levels for 35 days at 15 ± 0.5°C was not significantly different from that of the control group (Schiewe, 1974). One possible reason is that silver carps have strong tolerance to supersaturated TDG under non-lethal exposure, and when the TDG level does not exceed the tolerance threshold, fish can resist the stress of supersaturated TDG by self-adjustment (Ji *et al.*, 2021).

Under the lethal exposure of TDG supersaturation, the Ucrit of fish in this study decreased with exposure time at 11.0 ± 1.0°C but the differences among three TDG levels were not significant (Fig. 4C). Wang *et al.* (2018) found that the Ucrit of Prenant’s Schizothoracin (*Schizothorax prenanti*) decreased significantly at 23–25°C compared with the control group, but there were no significant differences in Ucrit among TDG levels (118%, 122%, 125% and 130%). Prenant’s Schizothoracin had the weakest tolerance to 130% at 24°C (Yuan *et al.*, 2018), and 120% TDG supersaturation is considered to be the tolerance threshold for Prenant’s Schizothoracin (Wang *et al.*, 2017). The study of Wang *et al.* (2018) can be regarded as a result of lethal exposure at an unfavorable water temperature, which was similar to the result of Ucrit of silver carps at 11.0 ± 1.0°C under lethal exposure. The water temperature of 11.0 ± 1.0°C was not optimum for silver carps, and high TDG (over 130%) had a negative effect on silver carps. As a result, the Ucrit of silver carps decreased with exposure time under lethal exposure at 11.0 ± 1.0°C.

The effect of supersaturated TDG on swimming performance under lethal exposure is usually negative (Wang *et al.*, 2017), while the effect of water temperature is positive (Guderley, 2004). It could be assumed that the increase or decrease of swimming performance mainly depends on which was dominant between water temperature and TDG supersaturation. When the water temperature was much lower than the optimum range for silver carps, the positive effect of water temperature is not enough to resist the negative effect of TDG. As a result, the Ucrit decreased significantly compared with the control group. The stress of supersaturated TDG was counteracted by the positive effect of water temperature when the water temperature was close to the optimum range. Thus, the Ucrit did not change significantly compared with the control group. Compared with the group of 11.0 ± 1.0°C, Ucrit of silver carps at 21.0 ± 1.0°C did not change significantly at the first 6 hours of lethal exposure time. We speculated that water temperature of 21.0 ± 1.0°C had a positive effect on the swimming performance of silver carps and counteracted the negative effect of supersaturated TDG, which finally resulted in a non-significant change in Ucrit of silver carps with lethal exposure time. However, with the increase of exposure time, the differences in Ucrit among TDG levels gradually appeared, which indicated that the compensation of water temperature became relatively weaker than the negative effect of TDG supersaturation.

## Limitation and implication

In the present study, our results showed that increasing water temperature could significantly improve the Ucrit of fish. However, only two temperature conditions were set and it might be not enough to comprehensively reflect the effect of water temperature on swimming performance. The present study focused on the effect of exposure mode on fish swimming ability, and it will be interesting to investigate the fish swimming performance in TDG supersaturated water with varying temperatures.

TDG supersaturation was not a stable condition and would be easily affected by high velocity and intensive turbulence in the swimming tunnel respirometer. Therefore, freshwater (100% TDG) was used in the swimming chamber during the Ucrit test period. It might provide a certain recovery for Ucrit although the duration for each test was restricted to 3 hours. Wang *et al.* (2015) found that after experiencing 130% TDG exposure, short term of freshwater recovery did not significantly extend the survive time of *S. prenanti*. So the results of Ucrit in this study might be a little larger than the true values considering the recovery factor during the swimming test but the differences might not be significant. Fish was fasted during the experiment, which may affect their swimming performance. Previous studies showed that 1 week of fasting did not have significant impact on the swimming performance of *Spinibarbus sinensis* and *Salmo salar* (Pang *et al.*, 2014; Hvas *et al.*, 2021). We think the impact of fasting in this study was also not significant.

This study indicated that there was no interaction between TDG level and exposure time on swimming performance of fish. The exposure time played a dominant role at lower temperature, while the TDG level had a significant impact at higher temperature under lethal exposure. It suggested that managers should increase the discharge flow rate to shorten the duration of flood discharge at lower temperature conditions although it may result in a higher TDG supersaturation. However, discharge flow rate should be decreased to maintain a relatively lower TDG supersaturation at higher temperature conditions for the conservation of pelagic species in the Yangtze River.

So far, there have been very limited studies focused on the effect of TDG supersaturation on the swimming performance of fishes in the Yangtze River basin. It is not enough for the ecological environment protection for the Yangtze River basin. Further field investigation on the migration behavior of fish under TDG supersaturation is urgently needed. The effect of TDG supersaturation caused by flood discharge on the life cycle of fish and other vulnerable species needs to be systematically evaluated. With the construction of large number of high dams on the Yangtze River basin, many fish species face the risk of supersaturated TDG stress. Different species or life stages have different tolerance to supersaturated TDG. This study only focused on the pelagic fish, and it is urgent to study the effect of supersaturated TDG on more vulnerable species to support the conservation of the water ecosystem in the Yangtze River basin.

## Conclusion

By systematically evaluating the swimming speeds of silver carps, the effects of TDG supersaturation level and exposure time at different temperatures on their Ucrit were studied. The following conclusions can be drawn:

(1) Under non-lethal exposure of TDG supersaturation, both the effects of TDG level and exposure time on the Ucrit of silver carps were not significant at different temperatures. However, there still existed a decreasing trend in Ucrit of silver carps with exposure time at lower water temperature.

(2) Under the lethal exposure of TDG supersaturation, the exposure time had a significant effect on the Ucrit of silver carps at lower water temperature, while the TDG level played a significant role at higher water temperature.

(3) For conditions with the same TDG supersaturation and exposure time, Ucrit in 21.0 ± 1.0°C water temperature was significantly greater than those in 11.0 ± 1.0°C.

## Funding

This research was funded by the National Natural Science Foundation of China (51809186, 52179075), the Key Science and Technology Program of Yunnan Province (Grant No.2019 BC002) and the China Postdoctoral Science Foundation (2019M663501).

## CRediT authorship contribution statement

Qianfeng Ji: Methodology, Resources, Writing—original draft, Writing—review and editing. Kefeng Li: Resources, Validation. Yuanming Wang: Conceptualization, Project administration, Funding acquisition. Ruifeng Liang: Formal analysis, Data curation, Funding acquisition. Jingjie Feng: Methodology, Resources. Ran Li: Investigation, Visualization. David Z. Zhu: Writing—review and editing.

## Data Availability Statement

Data are available from the authors upon reasonable request.

## Declaration of Competing Interest

The authors declare no conflicts of interest.
